# Prognostic significance of HLA-G in patients with colorectal cancer: a meta-analysis and bioinformatics analysis

**DOI:** 10.1186/s12885-023-11522-w

**Published:** 2023-10-24

**Authors:** Yingying Zhang, Siying He, Lisha Yu, Chao Shi, Yanyue Zhang, Shiyue Tang

**Affiliations:** 1 Department of clinical Laboratory, Affiliated Jinhua Hospital, Zhejiang University School of Medicine, Jinhua, China; 2 Department of Infectious Diseases, Affiliated Jinhua Hospital, Zhejiang University School of Medicine, Jinhua, China

**Keywords:** Colorectal cancer, HLA-G, meta-analysis, Prognosis

## Abstract

**Purpose:**

Human leukocyte antigen-G (HLA-G) has been reported to be aberrantly expressed in colorectal cancer (CRC); however, its prognostic value remains controversial. Hence, our meta-analysis aims to assess the prognostic value of HLA-G in CRC patients based on published literature and The Cancer Genome Atlas (TCGA) datasets.

**Methods:**

A systematic search was conducted on relevant studies retrieved from four electronic databases including PubMed, Embase, Web of Science and Cochrane Library. Hazard ratios (HRs) with 95% confidence intervals (CIs) were recorded to be applied as effective values. Fixed-effects models or random-effects models were applied on the basis of the value of heterogeneity (*I* ^2^). Publication bias was analyzed by Begg’s and Egger’s tests. In addition, the results were validated by using TCGA datasets.

**Results:**

Thirteen studies comprising 3896 patients were incorporated into this meta-analysis. The pooled results showed that HLA-G expression was significantly associated with poor overall survival (OS) in both the univariate analysis (HR = 1.44, 95% CI: 1.14–1.83, *P* = 0.002) and the multivariate analysis (HR = 1.55, 95% CI: 1.23–1.95, *P* < 0.001). Nevertheless, the expression of HLA-G is not related to age, sex, tumor type, tumor differentiation, TNM stage, or distant metastasis but lymph node metastasis. Notably, the prognosis of colorectal cancer was not consistent with the analysis result from TCGA data.

**Conclusion:**

HLA-G expression was significantly related to poor OS in CRC according to the results of our meta-analysis. However, we found that the prognostic significance was inconsistent with our results according to the TCGA data in CRC. Hence, more research is still needed to further illustrate the prognostic role of HLA-G in CRC.

## Introduction

Colorectal cancer (CRC) remains quite dangerous to human health. It ranks as the third most frequently diagnosed cancer in both males and females, and it is the second leading cause of cancer death, accounting for 9.5% of deaths worldwide [1]. Although the five-year survival rate of CRC has improved due to earlier detection or more advanced surgical techniques, some advanced-stage tumors with mutations still have a poor prognosis [2]. It has been reported that the five-year survival rate of CRC patients with oligometastatic disease through tumor resection and systemic treatment is 40%, while that of patients with metastatic CRC is only 14% [3, 4]. In addition, 30-50% of patients with CRC treated by curative resection are prone to recurrence [5]. Thus, it is essential to search for a novel biomarker to predict the survival time of CRC patients and serve as a new target for therapy.

The immune checkpoint human leukocyte antigen-G (HLA-G) is a non-classical HLA I molecule, which is able to induce immune tolerance. This molecule was confirmed by Geraghty in 1987 [6]. HLA-G was originally found in cytotrophoblasts [7], and is considered extremely important for fetal-maternal immunological tolerance [8, 9]. On the one hand, HLA-G is constitutively expressed on immunologically privileged tissue, such as the cornea, thymus, pancreatic islets, and endothelial cells. On the other hand, cancer, autoimmune disease, viral infection, inflammation and transplantation can induce HLA-G expression [10, 11]. Alternative splicing of the primary transcripts of HLA-G generates at least seven isoforms, namely, four membrane-bound (HLA-G1-HLA-G4) and three soluble (HLA-G5-HLA-G7) isoforms [12, 13]. These seven isoforms all contain the α1 domain. Since its discovery in melanoma in 1998 [14], HLA- G has been investigated extensively in a variety of carcinomas by an increasing number of scholars. HLA-G has immunomodulatory effects, which are mediated by binding to the ILT2/ILT4/KIR2DL4 receptor of immunocompetent cells or inducing the generation of regulatory T cells (Tregs) and myeloid-derived suppressor cells (MDSCs) [15]. In addition, HLA-G mediates immune tolerance via intercellular transfer pathways such as trogocytosis or exosomes [16]. Increasing studies have shown that HLA-G is related to the clinical parameters and prognosis of patients with different kinds of tumors [17]. Nevertheless, the prognosis of HLA-G expression in patients with CRC varies greatly. Ye et al. reported that HLA-G expression could lead to a shorter survival time in CRC patients [18]. However, Reimers et al. found that high HLA-G expression was significantly correlated with a better OS [19]. Therefore, this meta-analysis systematically evaluated the prognostic value of HLA-G in CRC patients.

## Methods

### Search strategy

This meta-analysis was performed according to the guidelines of the Preferred Reporting Items for Systematic Reviews and Meta-Analysis (PRISMA) [20]. We searched for relevant studies published until August 2023 in PubMed, Embase, Web of Science and the Cochrane Library. The search terms were as follows: (“HLA-G Antigens” OR “human leukocyte antigen G” OR “human leukocyte antigen-G” OR “HLA-G” OR “HLA G”) AND ((“colorectal neoplasms” OR “colonic neoplasms” OR “rectal neoplasms”) OR ((“colon” OR “colonic” OR “rectal” OR “rectum” OR “colorectal”) AND (neoplasm* OR neoplasms* OR cancer* OR cancers* OR tumor* OR tumors* OR carcinoma* OR neoplasia* OR neoplasias*))) AND (“prognosis” OR “prognostic” OR “prognoses” OR “outcome” OR “survival”). In addition, we evaluated the references and other relevant studies to identify more eligible publications.

### Inclusion and exclusion criteria

The eligible studies were identified by two independent authors. The inclusion criteria were as follows: (1) focused on humans; (2) assessed the relationship between HLA-G expression and survival outcomes; (3) provided hazard ratios (HRs) with corresponding 95% confidence intervals (CIs); and (4) included at least 100 patients with colorectal cancer.

The exclusion criteria were as follows: (1) duplicated articles; (2) reviews, meta-analysis, conference abstracts, case reports or letters; (3) animal models or cell lines as research subjects or studies concentrated on investigating mechanisms; or (4) lack of available survival data to obtain HRs and the associated 95% CIs.

### Data extraction and quality assessment

The relevant data from the included articles were reviewed and extracted independently by two researchers, and disagreements were resolved by discussion with a third author. The following information was collected: first author’s name, publication year, country, cancer type, clinicopathological parameters, number of patients, age, sex, expression, detection methods and outcomes (OS, DFS, CSS). Moreover, two investigators independently evaluated the quality of the eligible studies according to the Newcastle-Ottawa Scale (NOS, scores from 0 to 9); if the scores were ≥ 6, the studies were defined as high-quality articles.

### Validation by TCGA datasets

The data on HLA-G expression in colorectal cancer and corresponding normal tissues were obtained from Gene Expression Profiling Interactive Analysis (GEPIA, http://gepia.cancer-pku.cn) based on TCGA. The UALCAN (http://ualcan.path.uab.edu) was used to obtain the correlation between HLA-G expression and overall survival (OS). Kaplan-Meier Plotter (http://kmplot.com/analysis) was also used to further validate the results of UALCAN. In addition, the association between the expression levels of HLA-G and tumor-infiltrating lymphocytes (TILs) was analyzed by TISIDB (http://cis.hku.hk/TISIDB).

### Mechanism prediction of HLA-G

String (http://cn.string-db.org) was used to explore the related genes of HLA-G to obtain a protein-protein interaction network (PPI) of HLA-G. Furthermore, we conducted functional enrichment analysis of HLA-G-related genes, including Gene Ontology (GO) and Kyoto Encyclopedia of Genes and Genomes (KEGG) functional enrichment [21–23] by using DAVID (http://david.ncifcrf.gov), and the data were shown in a bar chart and bubble map plotted via https://www.bioinformatics.com.cn, an online platform for data analysis and visualization.

### Statistical analysis

The meta-analysis was performed using the Stata11.0 software (Stata Corporation, College Station, TX, USA). The pooled HR and corresponding 95% CIs were calculated to evaluate the prognostic value of HLA-G expression in colorectal cancer. Simultaneously, the correlation between HLA-G expression and clinical parameters was assessed by odds ratios (ORs) and corresponding 95% CIs. The chi-square test and *I* ^2^ test were used to assess the heterogeneity among the analyzed studies. A fixed-effects model was applied for analysis if the heterogeneity was not significant (*I* ^2^ < 50% or *P* > 0.05); otherwise, a random-effects model was adopted. Subgroup analysis was performed to identify the source of heterogeneity. Moreover, we conducted a sensitivity analysis to assess the stability of each independent article. Begg’s test and Egger’s regression were performed to evaluate potential publication bias. All statistical tests were two-sided, and *P* < 0.05 was considered statistically significant.

## Results

### Study selection and characteristics of the included research

**Fig. 1:**
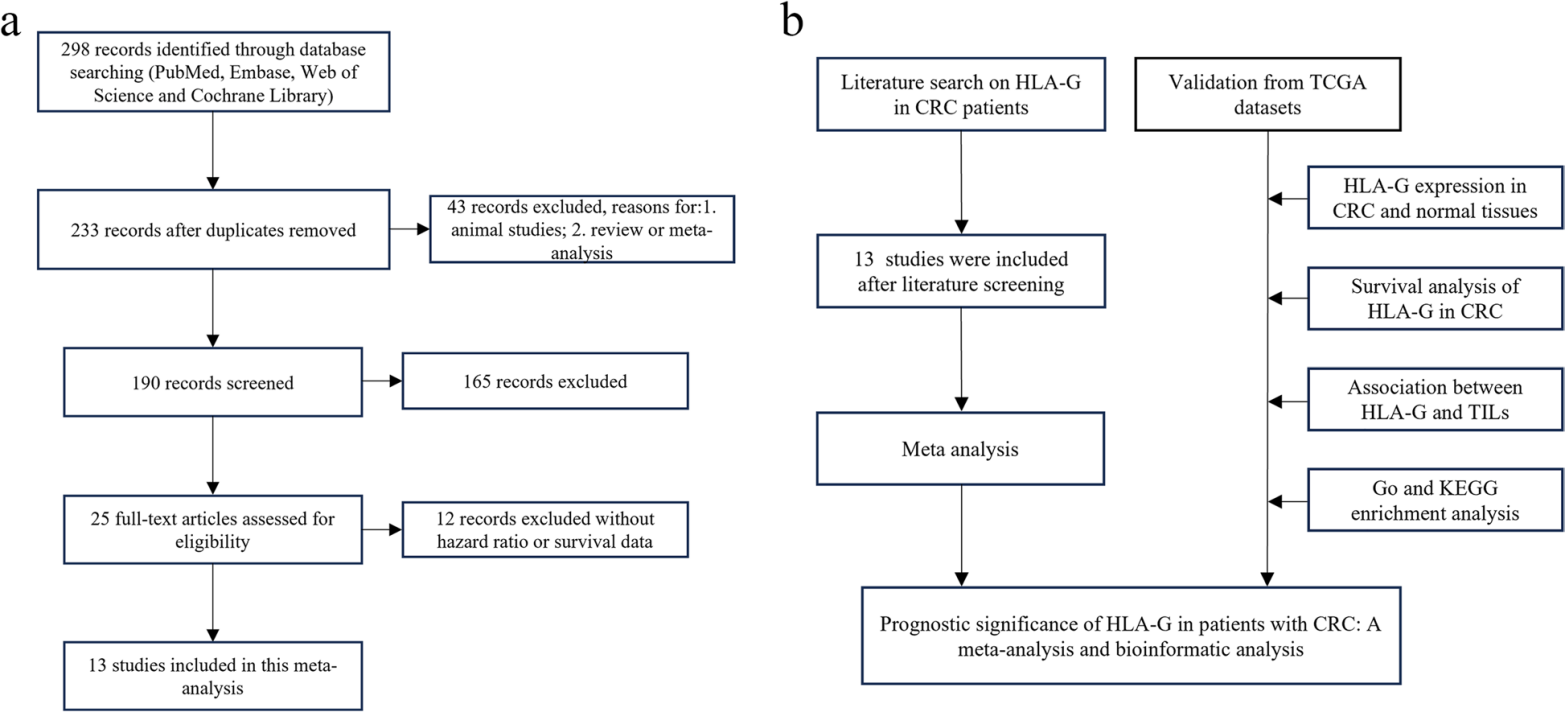
Schematic diagram of this study **a** Flow diagram of study selection process; **b** Flow diagram of the study

After a systematic search, 298 articles were retrieved from the four databases (PubMed, Embase, Web of Science and Cochrane Library). After exclusion of ineligible studies, 13 studies were included in this meta-analysis [18, 19, 24–34]. Among the 13 studies, 3 included patients with colon cancer only, 1 study looked at rectal cancer, and the remaining 9 were related to CRC. Figure 1 shows the detailed literature selection (Fig. 1a) and study process (Fig. 1b). The eligible studies were published between 2007 and 2022, during which a total of 3896 patients were enrolled and the prognostic value of HLA-G expression in CRC patients was evaluated. Immunohistochemistry (IHC) was used in almost all studies to evaluate the expression level of HLA-G, with only 2 studies using enzyme-linked immunosorbent assay (ELISA) and 1 using flow cytometry (FCM). The basic characteristics of the eligible articles are presented in Table 1.

**Table 1:** Main characteristics of the eligible studies in this meta-analysis

Study	Year	Country	Cancer type	No.of Patients	Age	Male	Method	Antibody	Pos vs Neg/High vs Low	Outcome	NOS
Ye et al [18]	2007	China	Colorectal cancer	201	64^m^	106	IHC	HGY	130/71	OS	7
Reimers et al [19]	2014	Netherlands	Rectal cancer	484	64.5^a^	316	IHC	4H84	134/350	OS,DFS	8
Zeestraten et al [24]	2014	Netherlands	Colon cancer	251	NA	137	IHC	4H84	51/200	OS,DFS	6
Guo et al [25]	2015	China	Colorectal cancer	102	NA	60	IHC	MEM-G/2	72/30	OS	6
Kirana et al [26]	2017	New Zealand	Colorectal cancer	254	NA	142	IHC	4H84	12/242	CSS	8
Li et al [27]	2017	China	Colorectal cancer	178	65^m^	100	ELISA	NA	89/89	OS	8
Zhang1 et al [28]	2017	China	Colorectal cancer	457	66^m^	268	IHC	4H84	323/134	OS	7
Zhang2 et al							IHC	4H84	299/158	OS	
Samadi et al [29]	2017	Iran	Colorectal cancer	100	50.52^a^	59	IHC	4H84	25/75	OS	6
Lin1 et al [30]	2018	China	Colorectal cancer	379	66^m^	214	IHC	4H84	268/111	OS	8
Lin2 et al							IHC	5A6G7	219/160	OS	
Jiao et al [31]	2020	China	Colorectal cancer	1037	62.3^a^	603	ELISA	NA	415/622	OS	9
Chen et al [32]	2021	China	Colorectal cancer	157	69.5^m^	89	FCM	MEM-G/9	72/85	OS	7
Bennedsen et al [33]	2022	Denmark	Colon cancer	188	71.5^m^	89	IHC	4H84	17/171	OS,DFS	8
Emirzeoglu et al [34]	2022	Turkey	Colon cancer	108	70^a^	60	IHC	4H84	45/63	OS,DFS,CSS	8

*m *median age, *a *average age, *IHC *Immunohistochemistry, *ELISA *Enzyme linked immunosorbent assay, *FCM *Flow cytometry, *NA *Not available, *Pos *Positive, *Neg *Negative, *OS *Overall survival, *DFS *Disease-free survival, *CSS *Cancer-specific survival, *NOS *Newcastle-ottawa scale

### Association of HLA-G expression levels with OS/DFS/CSS

**Fig. 2:**
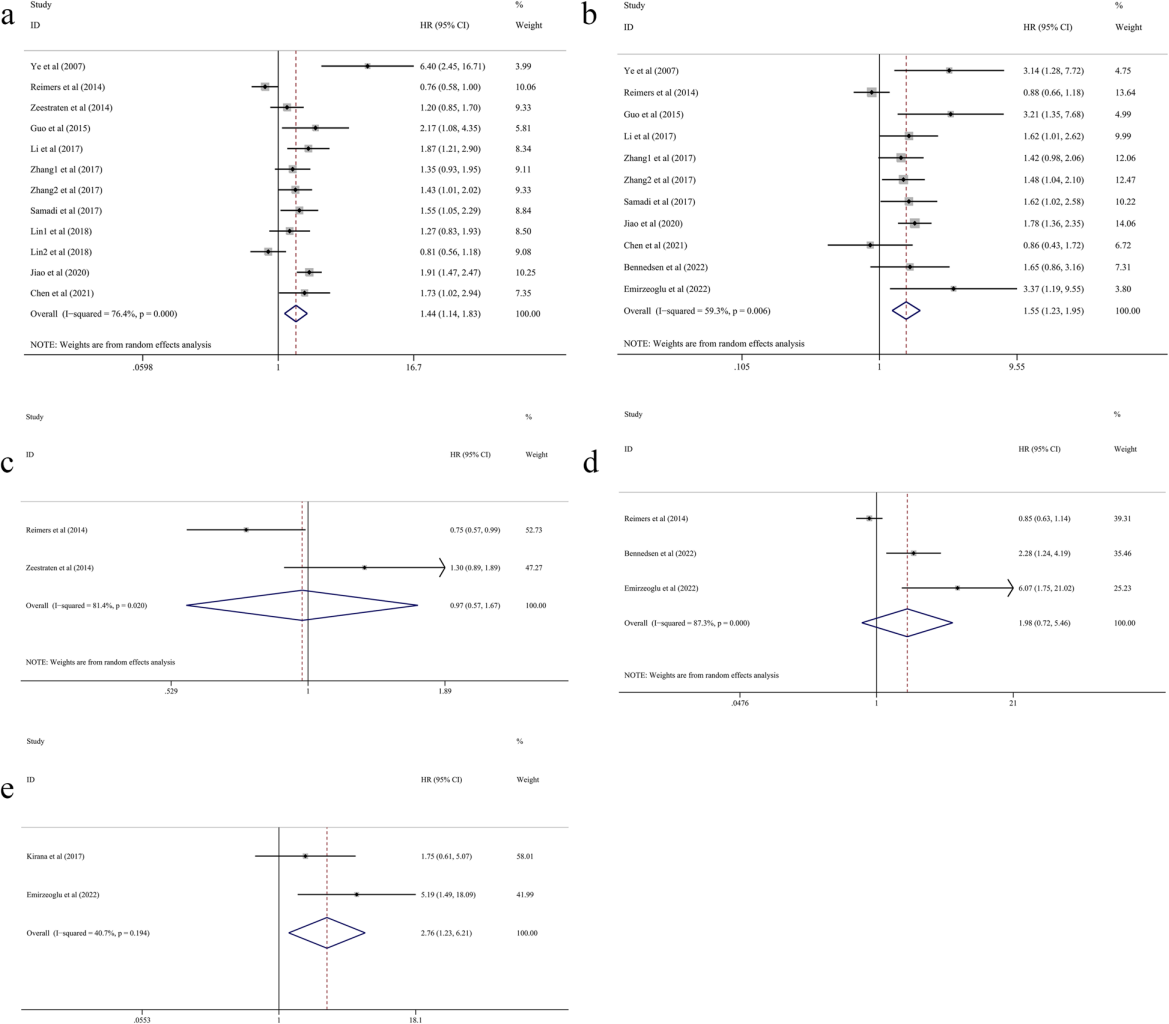
Forest plot of studies evaluating HRs of HLA-G expression and the prognosis of CRC patients. **a** HLA-G expression and the OS in the univariate analysis; **b** HLA-G expression and the OS in the multivariate analysis; **c** HLA-G expression and the DFS in the univariate analysis; **d** HLA-G expression and the DFS in the multivariate analysis; **e** HLA-G expression and the CSS in the multivariate analysis

Of the included articles, 12 studies (14 cohorts) involving 3642 patients were searched and screened to evaluate the association between HLA-G expression and overall survival. Ten articles (12 cohorts) involving 3346 patients were pooled for the univariate analysis, which showed that high HLA-G expression was significantly associated with poor OS (HR = 1.44, 95% CI = 1.14–1.83, *P* = 0.002), with significant heterogeneity (*I* ^2^ = 76.4%, *P* < 0.001) (Fig. 2a). Ten studies (11 cohorts) with 3012 patients were pooled for the multivariate analysis, which indicated that a high expression level of HLA-G was considerably correlated with poor OS (HR = 1.55, 95% CI = 1.23–1.95, *P* < 0.001) with remarkable heterogeneity (*I* ^2^ = 59.3%, *P* = 0.006) (Fig. 2b). Moreover, the pooled data of both the univariate analysis (HR = 0.97, 95% CI = 0.57–1.67, *P* = 0.920) (Fig. 2c) and multivariate analysis (HR = 1.98, 95% CI = 0.72–5.46, *P* = 0.186) (Fig. 2d) on DFS showed that HLA-G expression was not linked to DFS. The data from 2 studies (Kirana et al. [26] and Emirzeoglu et al. [34]) with 362 patients were pooled, and the results demonstrated that high HLA-G expression was associated with shorter CSS in patients with CRC (HR = 2.76, 95% CI = 1.23–6.21, *P* = 0.014) (Fig. 2e).

### Subgroup analysis for OS

**Fig. 3:**
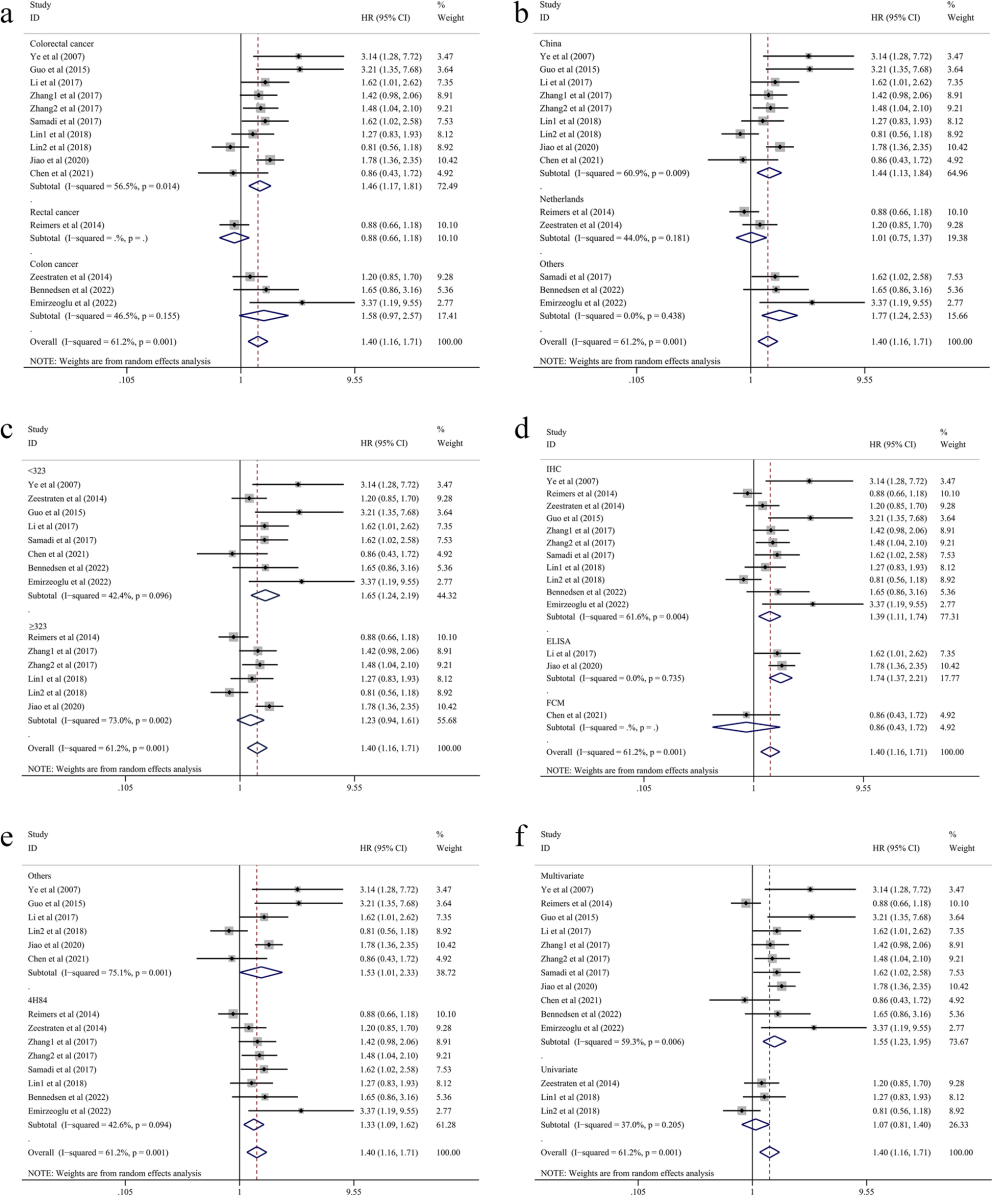
Forest plot for the subgroup analysis of OS. **a** cancer type; **b** country; **c** sample size; **d** method; **e** antibody; **f** analysis

The meta-analysis results showed that there was significant heterogeneity in the pooled HR of OS; therefore, a subgroup analysis of OS was performed to assess the association between the expression level of HLA-G and OS. Twelve articles with a total of 3642 patients were stratified into 6 groups based on cancer type, country, sample size, methods, antibody type and analysis method (Table 2). As a result, high HLA-G expression was significantly linked to shorter OS in CRC patients (HR = 1.46, 95% CI = 1.17–1.81, *P* = 0.001) and colon cancer patients (HR = 1.39, 95% CI = 1.04–1.86, *P* = 0.028) but not in patients with rectal cancer (HR = 0.88, 95% CI = 0.66–1.18, *P* = 0.418). Based on different detection methods of analysis, we found that the high expression of HLA-G was related to poor OS according to both immunocytochemistry (IHC) (HR = 1.39, 95% CI = 1.11–1.74, *P* = 0.005) and ELISA (HR = 1.74, 95% CI = 1.37–2.21, *P* < 0.001) results. Moreover, high HLA-G expression was associated with poor OS in other subgroups, such as patients from China (HR = 1.44, 95% CI = 1.13–1.84, *P* = 0.003), and a subgroup with a sample size < 323 (HR = 1.54, 95% CI = 1.26–1.87, *P* < 0.001); and the pooled HR of multivariate analysis (HR = 1.55, 95% CI = 1.23–1.95, *P* < 0.001) supported these results (Fig. 3).

**Table 2:** Subgroup analysis of pooled HR between HLA-G expression and OS

Subgroup	No. of studies	No. of cohorts	No. of patients	Pooled HR (95%)	*P* value	Heterogeneity	
				Fix/Random		*I* ^*2*^(%)	*P* value
OS	12	14	3642	1.40(1.16, 1.71)	0.001	61.2	0.001
Cancer type							
Colorectal cancer	8	10	2611	1.46(1.17, 1.81)	0.001	56.5	0.014
Rectal cancer	1	1	484	0.88(0.66, 1.18)	/	/	/
Colon cancer	3	3	547	1.39(1.04, 1.86)	0.028	46.5	0.155
Country							
China	7	9	2511	1.44(1.13,1.84)	0.003	60.9	0.009
Netherlands	2	2	735	1.00(0.80, 1.26)	0.984	44.0	0.181
Others	3	3	396	1.77(1.24, 2.53)	0.002	0.0	0.438
Sample size							
<323	8	8	1285	1.54(1.26, 1.87)	0	42.4	0.096
≥323	4	6	2357	1.23(0.94, 1.61)	0.129	73.0	0.002
Method							
IHC	9	11	2270	1.39(1.11, 1.74)	0.005	61.6	0.004
ELISA	2	2	1215	1.74(1.37, 2.21)	0	0.0	0.735
FCM	1	1	157	0.86(0.43, 1.72)	/	/	/
Antibody							
4H84	6	8	1967	1.27(1.10, 1.47)	0.001	42.6	0.094
Others	6	6	2054	1.53(1.01, 2.33)	0.047	75.1	0.001
Analysis							
Univariate	2	3	630	1.06(0.86, 1.32)	0.575	37.0	0.205
Multivariate	10	11	3012	1.55(1.23, 1.95)	0.000	59.3	0.006

*HR* hazard ratio, *OS *Overall survival, *IHC *Immunohistochemistry, *ELISA *Enzyme linked immunosorbent assay, *FCM *Flow cytometry

### Association of HLA-G expression and clinical parameters

To gain further insight into the value of HLA-G, we analyzed the correlation between the expression level of HLA-G and certain clinical parameters in CRC (Table 3). The outcome showed that high HLA-G expression was strongly linked to lymph node metastasis (HR = 1.20, 95% CI = 1.04–1.38, *P* = 0.010). Nevertheless, HLA-G expression was not related to other clinical parameters, such as age (HR = 0.95, 95% CI = 0.77–1.16, *P* = 0.593), sex (HR = 0.89, 95% CI = 0.76–1.04, *P* = 0.136), tumor type (HR = 1.18, 95% CI = 0.84–1.65, *P* = 0.340), tumor differentiation (HR = 0.71, 95% CI = 0.36–1.40, *P* = 0.320), TNM stage (HR = 1.05, 95% CI = 0.74–1.49, *P* = 0.798) or distant metastasis (HR = 1.42, 95% CI = 0.97–2.09, *P* = 0.072).

**Table 3:** Meta-analysis of the correlation between HLA-G expression and clinicopathological features of colorectal cancer

Clinicopathological parameters	studies	cohorts	No. of patients	OR (95% CI)	Significant Z	*P* value	Heterogeneity *I* ^*2*^(%)	*P* value	Model
Age (>66 vs ≤ 66)	2	4	836	0.95(0.77, 1.16)	0.53	0.593	0.0	0.828	fixed
Gender (male vs female)	10	12	2430	0.89(0.76, 1.04)	1.49	0.136	20.4	0.243	fixed
Tumor type (colon vs rectal)	7	8	2308	1.18(0.84, 1.65)	0.95	0.340	69.1	0.002	random
Tumor differentiation (moderate/well vs poor)	4	4	895	0.71(0.36, 1.40)	0.99	0.320	56.2	0.077	random
Lymph node metastasis (pos vs neg)	8	10	2695	1.20(1.04, 1.38)	2.56	0.010	49.6	0.037	fixed
TNM stage (III+IV vs I+II)	9	11	3122	1.05(0.74, 1.49)	0.26	0.798	78.3	0.000	random
Distant metastasis (pos vs neg)	6	8	2232	1.42(0.97, 2.09)	1.80	0.072	33.7	0.159	fixed

*pos *positive, *neg *negative

### Sensitivity analysis and publication bias

**Fig. 4:**
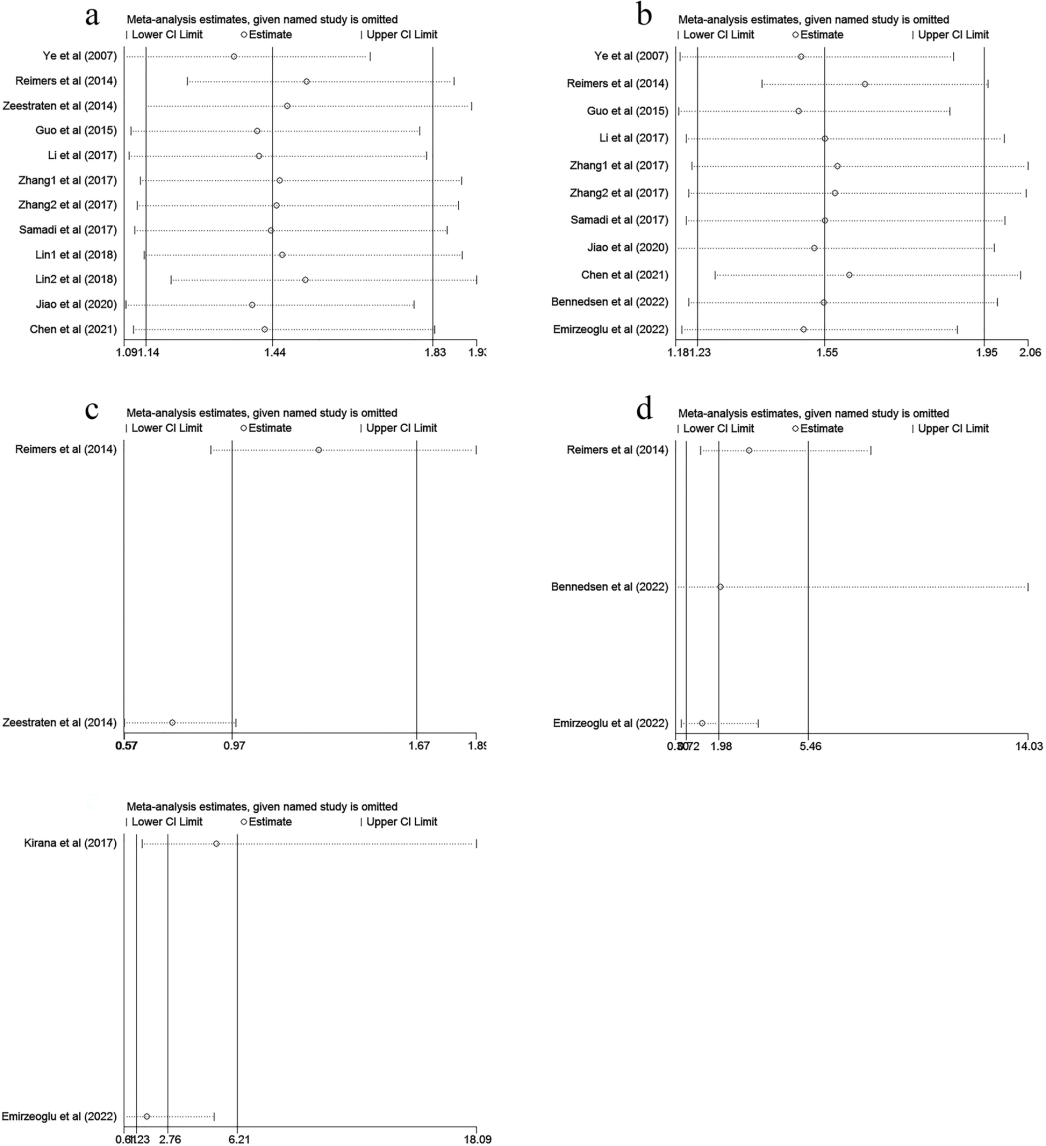
Sensitivity analysis for prognosis of CRC patients in this meta-analysis. **a** HLA-G expression and the OS in the univariate analysis; **b** HLA-G expression and the OS in the multivariate analysis; **c** HLA-G expression and the DFS in the univariate analysis; **d** HLA-G expression and the DFS in the multivariate analysis; **e** HLA-G expression and the CSS in the multivariate analysis

**Fig. 5:**
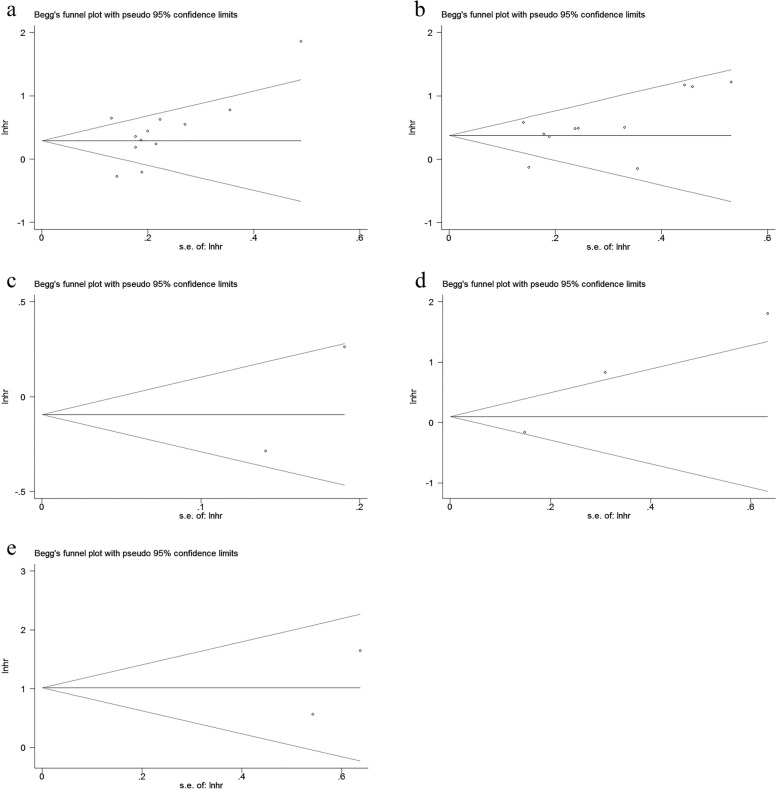
Begg’s funnel plots for assessing publication bias. **a** HLA-G expression and the OS in the univariate analysis; **b** HLA-G expression and the OS in the multivariate analysis; **c** HLA-G expression and the DFS in the univariate analysis; **d** HLA-G expression and the DFS in the multivariate analysis; **e** HLA-G expression and the CSS in the multivariate analysis

To evaluate the robustness of this meta-analysis, we carried out a sensitivity analysis to observe whether a single study could strongly affect the overall outcome. The results confirmed that excluding each eligible study had no impact on the pooled HR of OS, DFS and CSS (Fig. 4). Furthermore, Begg’s test and Egger’s regression test were performed to evaluate potential publication bias. We found that there was no publication bias in studies on HLA-G expression in terms of the association with OS, DFS and CSS (Fig. 5).

### Validation of the results from TCGA datasets

**Fig. 6:**
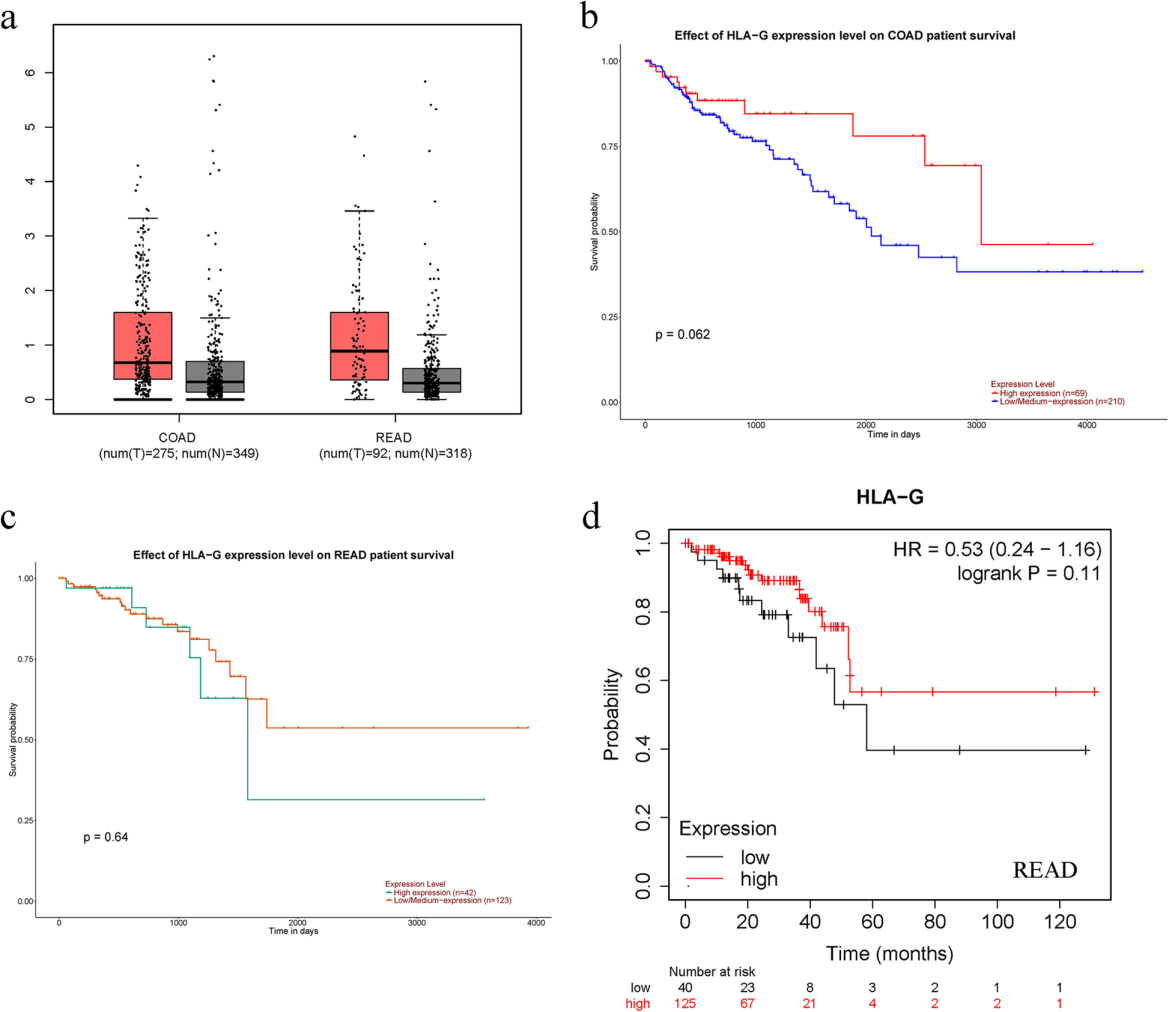
Verification of HLA-G expression and the prognostic value in TCGA database. **a** HLA-G expression in COAD and READ vs. normal tissue; **b** OS plot of HLA-G in COAD based on the UALCAN online database; **c** OS plot of HLA-G in READ based on the UALCAN online database; **d** OS plot of HLA-G in READ based on the Kaplan-Meier Plotter online database

**Fig. 7:**
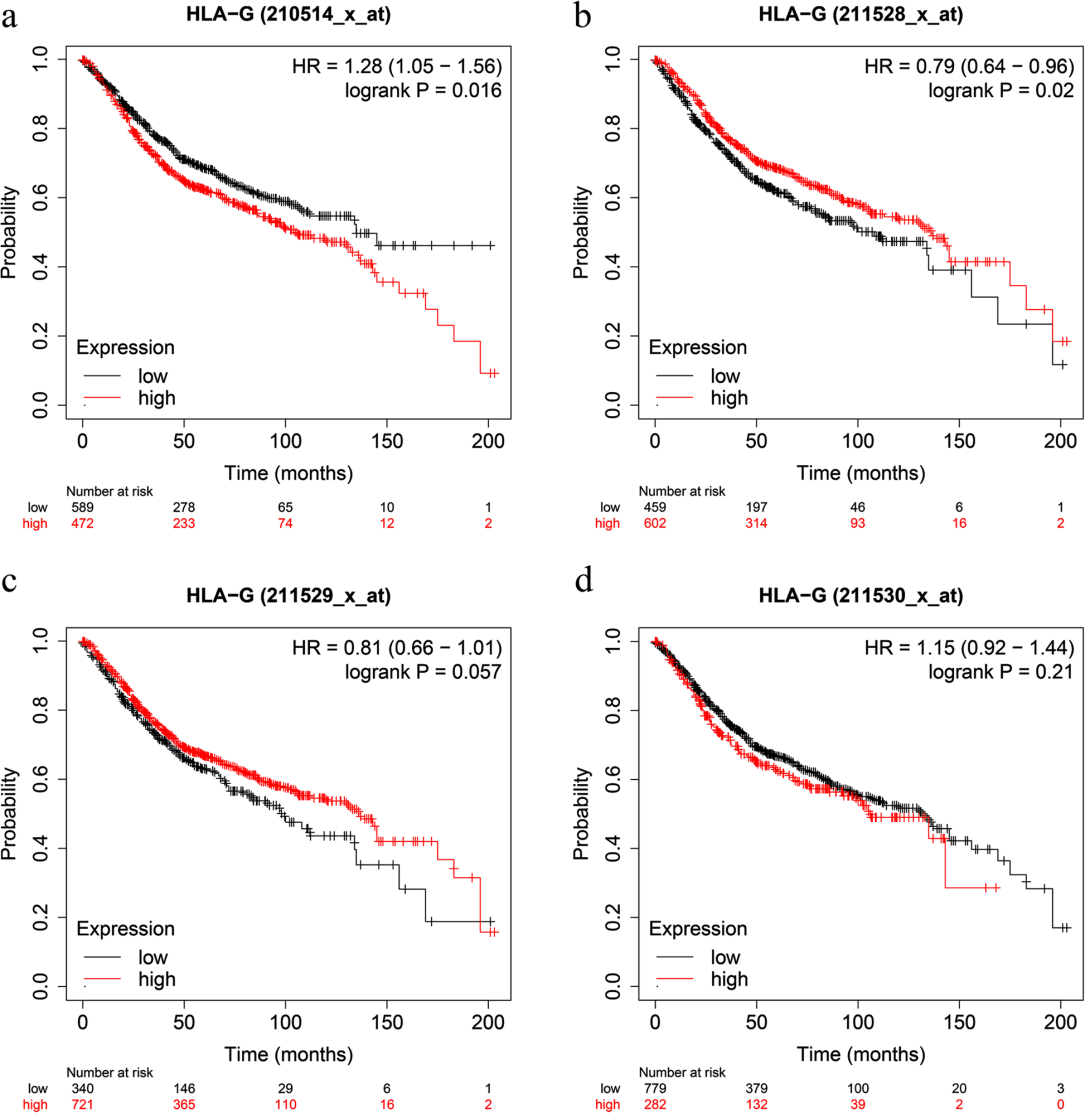
Verification of HLA-G expression and the prognostic value in patients with colon cancer based on the Kaplan-Meier Plotter online database. **a** OS plot of HLA-G in patients with colon cancer based on the 210514_x_at probe; **b** OS plot of HLA-G in patients with colon cancer based on the 211528_x_at probe; **c** OS plot of HLA-G in patients with colon cancer based on the 211529_x_at probe; **d** OS plot of HLA-G in patients with colon cancer based on the 211530_x_at probe

GEPIA, an online tool of visualized analysis based on TCGA and GTEx databases, was used to obtain the HLA-G expression of tumors and corresponding normal tissues. The expression of HLA-G in colon cancer or rectal cancer was higher than that in corresponding normal tissues, yet the difference was not significant (Fig. 6a). Moreover, UALCAN was applied to validate the prognostic value of HLA-G in CRC. The results revealed that there was no significant difference between HLA-G expression and OS in colon adenocarcinoma and rectum adenocarcinoma (Fig. 6b-c). In addition, we also used Kaplan-Meier plotter to confirm these results. Likewise, the results indicated that the expression level of HLA-G was not related to OS in rectal adenocarcinoma (READ) patients (Fig. 6d). However, the results in colon cancer patients were inconsistent based on different probes (Fig. 7a-d). Specifically, the data of the probe (Affy ID: 210514_x_at) displayed that high expression of HLA-G was significantly related to a worse prognosis (HR = 1.28, 95% CI = 1.05–1.56, *P* = 0.016) (Fig. 7a), which was consistent with our results. Nevertheless, the data of the 211528_x_at probe showed that high expression of HLA-G was associated with better OS in patients with colon cancer (HR = 0.79, 95% CI = 0.64–0.96, *P* = 0.02) (Fig. 7b). Moreover, the data showed that HLA-G (Affy ID: 211529_x_at and 211530_x_at) expression levels were not significantly correlated to the prognosis of patients (Fig. 7c-d).

**Fig. 8:**
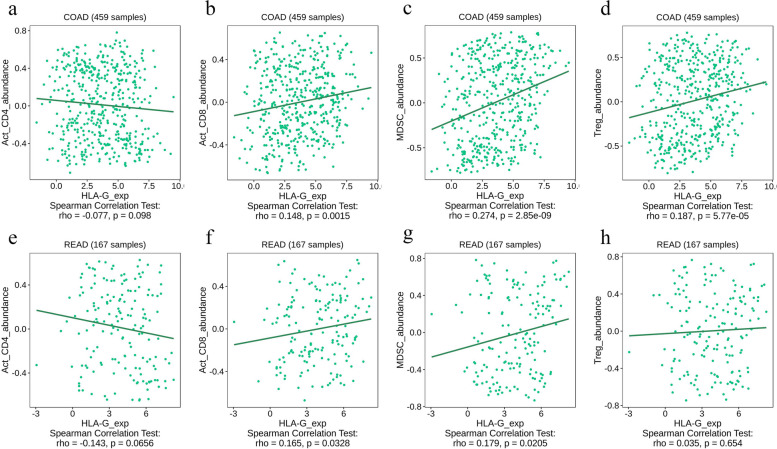
The correlation between HLA-G expression and tumor-infiltrating lymphocytes (TILs) in COAD and READ according to TISIDB. **a** activated CD4^+^ T cells in COAD; **b** activated CD8^+^ T cells in COAD; **c** MDSC in COAD; **d** Tregs in COAD; **e** activated CD4^+^ T cells in READ; **f** activated CD8^+^ T cells in READ; **g** MDSC in READ; **h** Tregs in READ.

Furthermore, TISIDB was used to investigate the correlation between HLA-G expression and tumor-infiltrating lymphocytes (TILs), including activated CD4^+^ T cells, CD8^+^ T cells, MDSCs and Tregs. The data revealed that HLA-G expression levels were negatively associated with the levels of activated CD4^+^ T cells, however, the difference was not statistically significant (Fig. 8a and e). In addition, activated CD8^+^ T cells, MDSCs and Tregs were expanded with increasing expression levels of HLA-G in colon cancer and rectal cancer (Fig. 8b-d and f-h).

### PPI network construction and functional enrichment analysis

**Fig. 9:**
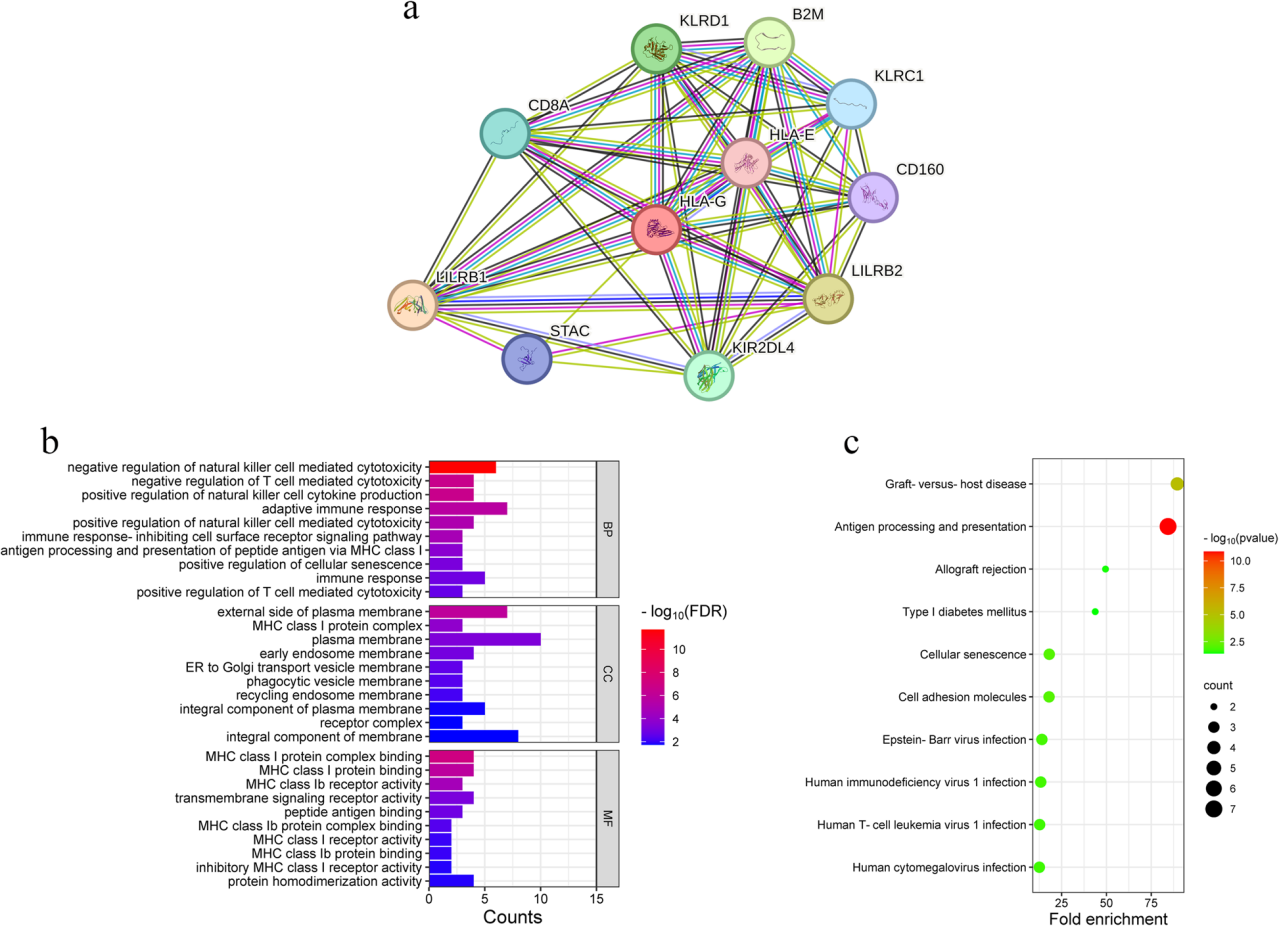
Mechanism prediction of HLA-G-related genes with bioinformatics. **a** The protein-protein interaction network of HLA-G; **b** GO enrichment analysis of HLA-G-related genes; **c** KEGG enrichment analysis of HLA-G-related genes

We performed a PPI network analysis of HLA-G-related genes by using STRING, and the PPI network involved 11 nodes and 49 edges (Fig. 9a). Moreover, the HLA-G-related genes were used for functional enrichment analysis via DAVID. GO terms included biological process (BP), cell components (CC) and molecular function (MF). The data indicated that in the BP aspect, HLA-G and its related genes were mainly enriched in adaptive immune response, negative regulation of natural killer cell mediated cytotoxicity, immune response and negative regulation of T cell mediated cytotoxicity. In the CC aspect, these HLA-G-related genes were significantly enriched in plasma membrane, integral component of membrane, external side of plasma membrane and integral component of plasma membrane. In the MF aspect, MHC class I protein complex binding, transmembrane signaling receptor activity, protein homodimerization activity and MHC class I protein binding were major GO terms (Fig. 9b). In addition, KEGG pathway analysis was conducted (Fig. 9c), and the HLA-G-related genes were mainly enriched in antigen processing and presentation and graft-versus-host disease.

## Discussion

CRC is a common malignant carcinoma of the gastrointestinal tract, and is regarded as a great challenge to public health worldwide. According to the latest statistics, there are approximately 147,950 new cases of CRC, and 53,200 individuals deaths from the disease, with approximately 12% of new cases diagnosed and 3640 deaths occurring in adults aged younger than 50 years [2]. Traditional therapy, targeted therapy and immunotherapy are the main effective therapeutic strategies to improve the survival time of patients with CRC [35]. Immunotherapy has been applied to all kinds of solid and hematological malignancies, particularly immune checkpoint inhibitors (ICIs) [36]. Immune checkpoints, a class of molecules expressed by immunocompetent cells, such as cytotoxic T lymphocyte antigen 4 (CTLA4) and programmed cell death-1 (PD-1), participate in and regulate immune activation [37]. Nevertheless, a majority of colon cancer patients develop drug resistance during immunotherapy [38]. Therefore, it is necessary to explore new therapies in CRC patients by means such as searching for novel molecular markers.

Currently, HLA-G is considered as a novel immune checkpoint that is neo-expressed in tumor cells and promotes tumor immune escape [10]. A large amount of evidence has revealed that HLA-G is strongly linked to tumor angiogenesis, escape, metastasis and survival of patients [39]. However, the prognostic role of HLA-G in CRC among published articles is ambiguous. Hence, we screened relevant studies and performed this meta-analysis to further investigate the potential prognostic value of HLA-G expression.

A total of 13 eligible studies with 3896 patients were included in our meta-analysis. Our results indicated that the HLA-G expression level was related to poor OS and CSS in CRC. A meta-analysis published in 2021 showed that HLA-G expression was correlated with a poor prognosis in gastrointestinal (GI) cancer patients [40], and recently, according to the report of Bartolome et al. [41], HLA-G expression was higher in colorectal cancer (HR = 1.55, 95% CI = 1.16 - 2.07), which was consistent with our results. In addition, we further investigated the correlation between HLA-G expression and the clinicopathological parameters of CRC patients. It was obvious that HLA-G expression was significantly correlated with lymph node metastasis. Moreover, subgroup analysis revealed that the main sources of heterogeneity were generated by cancer type, race, detection methods, sample size and analysis method. Zhang et al. reported that HLA-G could be considered a prognostic factor for CRC patients when the cut-off value of HLA-G expression was 55%, while when the cut-off value was 5%, HLA-G expression was not related to the survival time of CRC patients [28]. HLA-G expression detected with the monoclonal antibody (mAb) 4H84 in lung cancer was significantly related to disease stage and a poor prognosis, while this association was not observed when using the mAb 5A6G7 [42, 43]. Furthermore, Lin et al. [30] demonstrated that the expression levels of HLA-G detected with the mAbs 4H84 and 5A6G7 in CRC patients were distinct, which indicated that there were potential HLA-G isoforms in CRC, which was consistent with the report of Tronik-Le Roux et al. [44]. Additionally, they unexpectedly found that there was a potential unidentified HLA-G isoform and its independent clinical relevance. We concluded that it is necessary to establish uniform standard methods for HLA-G detection and evaluation. Furthermore, the commercial reagents of anti-HLA-G antibodies recognize the α1 domain or intron 4, which leads to certain limitations for detecting HLA-G. Moreover, heterogeneity also plays a crucial role in HLA-G expression; this includes intra-tumor and inter-tumor heterogeneity, which may contribute to controversial results. Thus, there is still more work to be performed to explore the unidentified HLA-G isoforms, and construct uniform standards to better research the prognostic value of HLA-G in CRC patients.

GEPIA was used to obtain the expression of HLA-G in tumor tissues and corresponding normal tissues. The bioinformatic results showed that there was a nonsignificant difference, even though HLA-G expression in colon cancer or rectal cancer was higher than that in their corresponding normal tissues. We also used UALCAN and Kaplan-Meier Plotter to generate the survival curves based on HLA-G expression for CRC patients from the TCGA database. Notably, the results were not completely consistent with our meta-analysis results. In the Kaplan-Meier Plotter, the data of the 210514_x_at probe was consistent with our results, while it could be seen that high expression of HLA-G was significantly associated with better OS in colon cancer patients from the 211528_x_at probe. Because, these probes, corresponding to different sequences of the target gene HLA-G, were designed for different HLA-G isoforms. It is acknowledged that HLA-G has at least seven isoforms due to alternative splicing of primary transcript, and up to now, specific antibodies mainly focus on HLA-G1 and HLA-G5. Thus, it is possible that different HLA-G isoforms have their unique clinical prognostic significance, which need to be further explored. In addition, the process by which the gene affects HLA-G protein expression may be influenced by transcriptional regulation, post-transcriptional processing and post-translational modification. Polymorphic sites could affect the stability of HLA-G mRNA and the affinity of microRNA to bind to HLA-G, which may contribute to the levels of HLA-G expression. HLA-G polymorphisms mainly involve 5’-upstream regulatory region (5’URR) polymorphisms and 3’-untranslated region (3’UTR) polymorphisms [45]. It has been reported that the soluble HLA-G (sHLA-G) levels in the peripheral blood with the 14 bp Del allele were higher than those in sHLA-G with the 14 bp Ins allele [46–48]. In addition, there was a research reported that overexpressed miR-365 under hypoxic conditions could target the 3’UTR of the HLA-G mRNA to suppress its expression [49]. Accumulating evidence has shown that long non-coding RNAs (lncRNAs) could be novel biomarkers to predict the prognosis of patients with cancer [50]. According to Wang et al.’s report [51], lncRNA myocardial infarction associated transcript (MIAT) was correlated with poor OS, and it was noted that MIAT and miR-133 participated in the proliferation and metastasis of pancreatic cancer [52]. A previous study reported that miR-133 could regulate HLA-G expression by binding to the 3’-UTR [53]. We speculate that MIAT/miR-133 might be involved in the regulation of HLA-G expression in CRC. Moreover, a study showed that low DNA methylation levels could lead to HLA-G overexpression [54]. Bucova et al found that methylated MGMT promoters in patients with glioma had lower sHLA-G levels than unmethylated MGMT promoters [55]. Therefore, the regulation of HLA-G expression levels involves complicated mechanisms that need to be further explored.

Studies on the mechanism of HLA-G in CRC are limited. Increasing evidence has revealed that TILs play a pivotal role in the prognosis of patients with CRC [56, 57]. Hence, TISIDB was used to obtain the correlation between HLA-G expression and TILs in CRC patients. The data showed that activated CD4^+^ T cells levels were decreased with HLA-G expression. Bainbridge et al. demonstrated that HLA-G could inhibit CD4^+^ T cells proliferation [58]. It was also shown that HLA-G expression was positively correlated with the levels of activated CD8^+^ T cells in patients with COAD and READ. Additionally, HLA-G expression was significantly positively related to MDSCs and Tregs. Previous studies demonstrated that HLA-G could promote the proliferation and activation of MDSCs and Tregs [59, 60]. It is widely acknowledged that increased circulating MDSCs in patients with late-stage cancer are associated with tumor progression and metastasis [61]. Therefore, HLA-G plays a crucial role in the tumor microenvironment by inhibiting the functions of effector cells and increasing MDSCs as well as Tregs. Moreover, we performed functional enrichment analysis to investigate the function of HLA-G and its co-expressed genes. The data revealed that HLA-G and its related genes were significantly enriched in the negative regulation of NK cells and T cells mediated cytotoxicity.

It is well known that HLA-G is ectopically expressed in various kinds of cancers, participating in tumor progression and patient survival [39]. A growing body of evidence suggests that HLA-G, a potential novel immune checkpoint, is a promising target for immunotherapy. Recently, Zheng et al. suggested that HLA-G/KIR2DL4 signaling provided novel insights into trastuzumab resistance in breast cancer [62]. Morandi et al. envisaged that the combination of anti-HLA-G antibodies with other immune checkpoints could be a novel immunotherapy to improve the clinical outcome of patients [63]. Furthermore, preclinical research on anti-HLA-G antibody therapy in non-muscle-invasive bladder cancer was developed [64]. One study reported that the HLA-G inhibitor TTX-080 was being used in patients with advanced solid cancer in an early clinical trial [65]. These clinical trials suggest that the HLA-G antibodies are promising for the clinical treatment of CRC.

Our meta-analysis is the first to validate the association between HLA-G expression and prognosis in patients with colorectal cancer using bioinformatics. The possible roles of HLA-G in TILs were also studied. However, there are several limitations in our meta-analysis. First, it is inevitable that unpublished articles and non-English literature were not mentioned in our meta-analysis; additionally, some publications lacking HR and 95% CI data were also excluded. Second, given the small numbers of articles included, there was no uniform method used for the detection of HLA-G, such as IHC, ELISA or FCM, which may generate potential heterogeneity. Third, it is well known that HLA-G contains at least seven isoforms that are four membrane-bound (HLA-G1-HLA-G4) and three soluble (HLA-G5-HLA-G7) isoforms, and distinct HLA-G isoforms may have unique biological functions as well as clinical significance. Thus, the chosen anti-HLA-G antibodies may influence the results of this meta-analysis. Fourth, the definition of cut-off values of HLA-G lacks a uniform standard, which may result in heterogeneity. Finally, given that the number of CRC patients with available data on HLA-G expression and clinical parameters is small, more studies are still needed to confirm these results.

## Conclusion

In summary, our meta-analysis showed that HLA-G expression was associated with lymph node metastasis and poor OS in CRC patients, yet the bioinformatics analysis showed that the prognosis of HLA-G was inconsistent. Notably, HLA-G was correlated with the levels of TILs, including CD4^+^ T cells, CD8^+^ T cells and MDSCs, which suggested that HLA-G plays an important role in the tumor environment. HLA-G is a promising prognostic biomarker for CRC patients and may provide novel insight into the immunotherapy in CRC. In addition, more investigations are still needed to demonstrate the prognostic value of HLA-G in CRC.

## Data Availability

The datasets generated and/or analysed during the current study are available from the corresponding author on reasonable request.

## Abbreviations

HLA-G: Human leukocyte antigen-G
CRC: Colorectal cancer
TCGA: The Cancer Genome Atlas
OS: Overall survival
Tregs: Regulatory T cells
MDSCs: Myeloid-derived suppressor cells
ELISA: Enzyme-linked immunosorbent assay
FCM: Flow cytometry
DFS: Disease-free survival
CSS: Cancer-specific survival
CTLA4: Cytotoxic T lymphocyte antigen 4
PD-1: Programmed cell death-1
COAD: Colon adenocarcinoma
READ: Rectal adenocarcinoma
ccRCC: Clear-cell renal cell carcinoma
